# Case report: Surgical treatment of a primary giant epithelioid hemangioendothelioma of the spine with total en-bloc spondylectomy

**DOI:** 10.3389/fonc.2023.1109643

**Published:** 2023-03-28

**Authors:** Wanbao Ge, Yuan Qu, Tingting Hou, Jiayin Zhang, Qiuju Li, Lili Yang, Lanqing Cao, Jindong Li, Shanyong Zhang

**Affiliations:** ^1^ Department of Spine Surgery, Second Affiliated Hospital of Jilin University, Changchun, Jilin, China; ^2^ Department of Pathology, Second Affiliated Hospital of Jilin University, Changchun, Jilin, China; ^3^ Department of Thoracic Surgery, Second Affiliated Hospital of Jilin University, Changchun, Jilin, China

**Keywords:** epithelioid hemangioendothelioma, total en-bloc spondylectomy, vertebral lesion, vascular neoplasm, giant

## Abstract

**Background:**

Epithelioid hemangioendothelioma (EHE) is an extremely uncommon malignant neoplasm that originates from vascular endothelial or pre-endothelial cells. In this report, we present the case of patient who was diagnosed with a primary giant EHE of the spine and underwent treatment with total en-bloc spondylectomy (TES).

**Case presentation:**

A 43-year-old male patient with a history of he presented to our hospital with chronic and progressive back pain. Physical examination revealed weakened sensation of acupuncture and touch on the left costal arch, while relatively normal neurological functions were preserved. Radiological examinations identified a giant destructive soft tissue lesion occupying the T8 vertebral region, with moderate destruction of the pedicle and lamina, as well as the 7^th^ left rib. A preoperative biopsy of the 8th vertebra resulted in a diagnosis of epithelioid hemangioendothelioma(EHE). Postoperative immunohistochemical and pathological reports confirmed the presence of EHE in the left ribs and T8 ribs. The patient underwent resection of the 7th left rib and posterior pedicle screw fixation with 8 pairs of screws and a titanium mesh cage. Subsequently, thoracic en bloc spondylectomy was performed on the T8 vertebra. The patient did not receive radiation or chemotherapy following surgery. Over a period of 3 years, the patient remained free of disease and relapse.

**Conclusion:**

The use of transarterial embolization with spherical embolic agents (TES) has been demonstrated to be a safe, effective, and reliable treatment option for hepatic epithelioid hemangioendothelioma (EHE). Nevertheless, it is crucial to conduct long-term follow-up of this patient in order to assess their clinical outcome.

## Introduction

Epithelioid hemangioendothelioma (EHE) is an exceedingly uncommon tumor that originates from vascular endothelial or pre-endothelial cells. This neoplasm exhibits histological properties that fall in between those of hemangiomas and high-grade angiosarcomas. Although EHE can manifest in any organ of the body, it is most frequently observed in the liver, lung, and skin, but rarely affects the spine (1, 2). Epithelioid hemangioendothelioma (EHE) has a prevalence of one in a million (3). Although mortality is generally low, some cases can lead to death due tometastases (4). Atypical localized pain is the most frequent early symptom of EHE, which serve as an indication of the disease that can later progress into a chronic illness (5). Consequently, the exclusion of other diseases in patients with primary spinal tumors can occasionally lead to an accidental diagnosis of EHE.

Despite its relatively low mortality, delayed diagnosis and treatment of EHE can result in a protracted and potentially fatal illness. Nonetheless, we report a case of primary giant EHE of the spine in which the patient underwent early wide-margin resection without postoperative adjuvant therapy. As a result, the patient achieved long-term survival free from disease and without any instrument-related complications.

## Case report

A 43-year-old man presented to our hospital on foot with chronic and progressive back pain. He had been experiencing paroxysmal back pain for approximately 1 year., which was more pronounced at night and not relieved by rest. In the 15 days leading up to his admission, the back pain had intensified, accompanied by mild radiating pain and numbness in his lower limbs. The patient did not report any other physical discomfort, except for the pain, and denied any disease-related complaints. Upon further inquiry, he reported a slight weight over the past month. The patient had no significant strong family medical history of cancer or congenital diseases.

Upon physical examination, the patient exhibited weakened sensation to fine-touch and pinprick along his left costal arch, as well as grade 4 strength in his lower extremities. Pressure and percussion pain were observed over the T8 vertebra. However, the straight-leg raising test and the Babinski sign were bilaterally negative. The patient’s bilateral deep tendon reflexes for the Achilles tendon and knee jerk reflexes were normal. And no anomalies in the spinal cord or neurological conditions in the upper extremity were noted. Nonetheless, the patient exhibited evident anxiety and a strong desire for surgery.

Diagnostic tests were conducted to identify cancer cell markers, and routine laboratory tests were performed to determine the patient’s condition. The laboratory results were almost within the normal range, which increased uncertainty regarding the diagnosis of EHE, except for the elevated levels of carcinoembryonic antigen alpha-fetoprotein was elevated (16.81 ng/ml, normal: <8.78 ng/ml)and cytokeratin 19 fragment Cyra21-1 (2.74 ng/ml,normal: <2.08 ng/ml). A thoracic magnetic resonance imaging (MRI) revealed a vertebral lesion, which was assessed to determine the degree of flexibility of the vertebral spine, and a surgical intervention approach was finalized as the treatment course.

**Figure 1:**
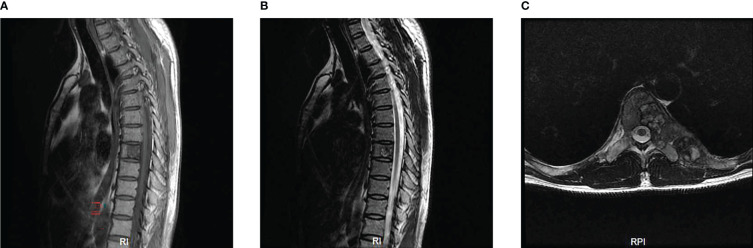
Preoperative MRI scan of the patient’s thoracic spine **(A–C)**. Sagittal scan revealed the density of obvious bone destruction in the T8 on T1 and T2-weighted images **(A, B)**. Transverse MRI scan showed a 6.5cm x 2.0cm sized and well-defined lesion occupying the T8 vertebral body, pedicle and lamina **(C)**.

**Figure 2:**
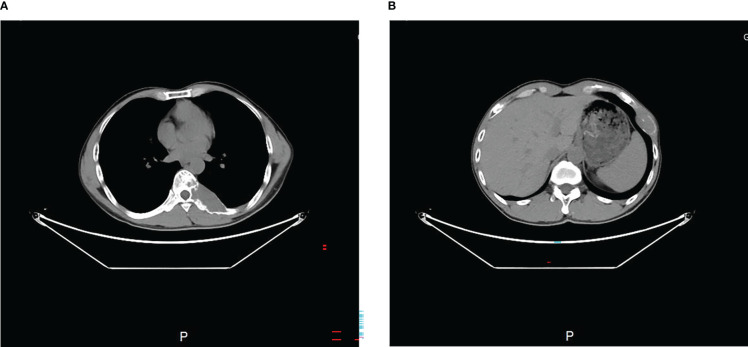
Preoperative transverse CT scan **(A, B)** showed the tumor occupied the 8th thoracic vertebral body and 7th rib.

The MRI showed a giant destructive soft tissue lesion occupying the T8 vertebral region with moderate destruction of the pedicle and lamina (**Figures 1A, B**). Another lesion was found on the seventh left rib (**Figure 1C**). The preoperative medical tests, including an electrocardiogram and chest CT scan (**Figure 2**).

**Figure 3:**
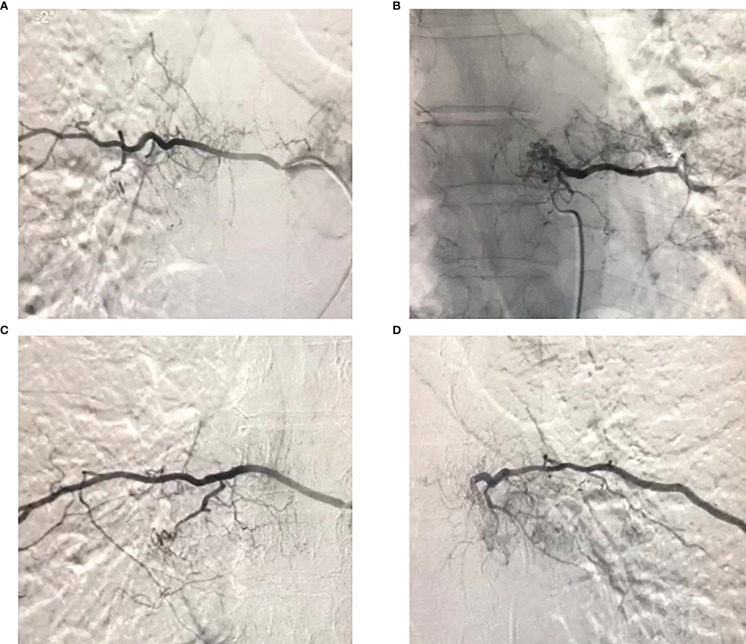
Preoperative angiography of the 7th and 8th thoracic vertebral segment arteries showed that the tumor had a rich blood supply, and embolization was performed.

**Figure 4:**
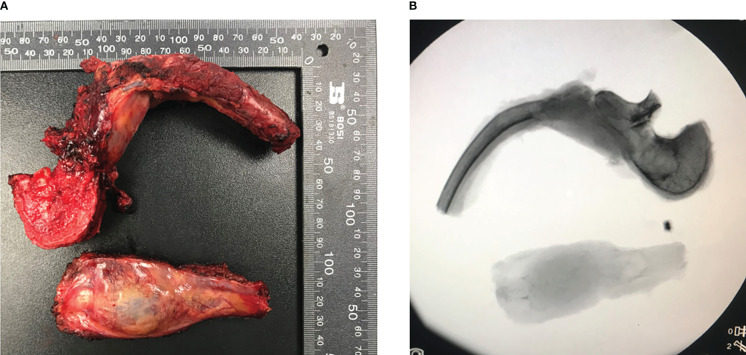
En-bloc excision tumor specimen in T8 and part of 7th rib of the patient and corresponding perspective image **(A, B)**. Both images showed the integrity of tumor and the lesion was completely removed.

Previous studies have recommended preoperative embolization to generally “shrink” the tumor (5). Therefore, vascular embolization was planned and performed. Preoperative angiography showed abundant blood supply in the tumor (**Figure 3**). To decrease intraoperative blood flow volume and maintain vital signs, four segmental arteries were embolized 12 hours preoperatively. EHE was ultimately diagnosed by a standard biopsy to determine the therapy course. Neuroelectromyography testing was not performed and an IV infusion of cephalosporin was administered 30 minutes before surgery to prevent infection. Tranexamic acid was not administered for prophylactic hemostasis. In consultation with a thoracic surgeon and an anesthesiologist, resection of the 7th rib and total en-bloc spondylectomy (TES) were performed sequentially. The patient was positioned right lateral decubitus and the rib was resected. During costectomy, a mass measuring 4.5 × 11 cm could be recognized, and no implant nodules, hydrothorax, or visible abnormality in the lungs were found around the 7th rib (**Figure 4A**). The patient was then transferred to prone location of TES. During TES, the pedicles of T6, T7, T9, and T10 were fixed with routine screws first (Screws are made of titanium alloy. Lengths are T6:35mm, T7:35mm, T9:35mm, T10:40mm, respectively. Diameters are T6:40mm, T7:40mm, T9:40mm, T10:45mm, respectively). C-arm fluoroscopy showed satisfactory positioning of the pedicle screws bilaterally. The left 8th vertebral body was exposed, and a giant tumor invading the vertebral body and the costovertebral joint was resected (**Figure 4B**). An appropriate titanium mesh cage was implanted and the incision was closed with drains. The whole surgical procedure was successful, and the intraoperative blood loss was only about 1000 ml. The proper placement of the surgical implants was immediately checked by X-ray.

**Figure 5:**
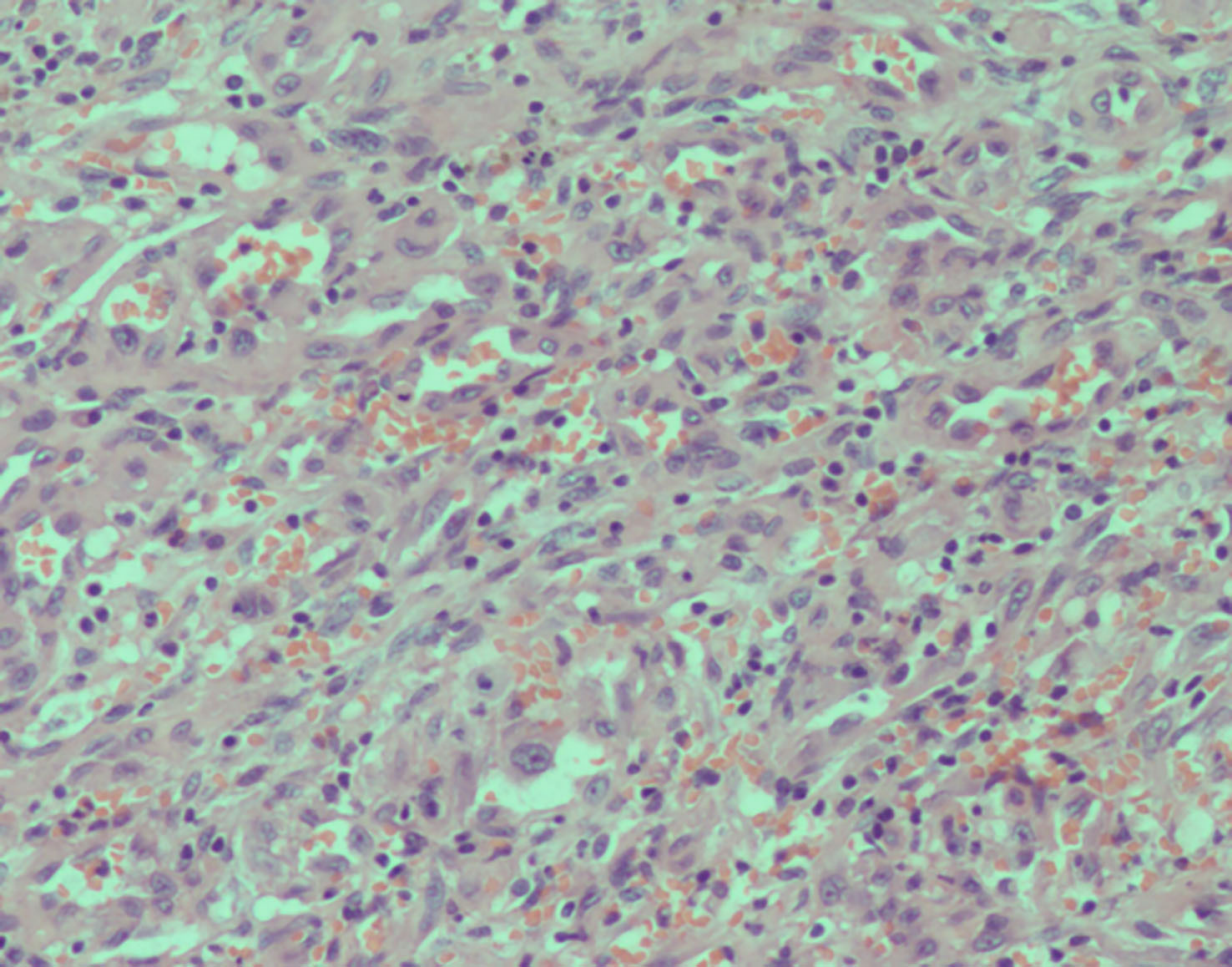
Histopathology of the resected mass. High-power magnification (200×) shows cords and groups of epithelioid cells in a dense without well-formed vascular structures.

**Figure 6:**
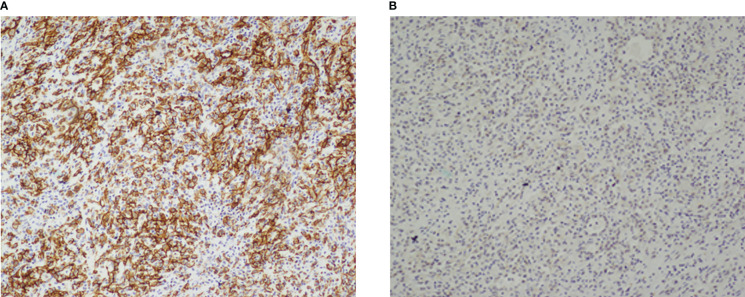
Immunohistochemical stain of the resected mass. **(A, B)** Tumor cells expressed CD31 **(A)** and FIL-1 **(B)**. Both immunohistochemical stain were in 100×magnification.

Postoperative pathology reports confirmed the diagnosis of EHE. Microscopic examination revealed that mostly eosinophilic endothelial cells constituted the tumor, without any well-formed vessels (**Figure 5**). Immunohistochemical staining was performed for friend leukemia integration 1 (FLI-1), CD31, and CD34 expression. Combined CD31 and FLI-1 staining suggested the diagnosis of EHE (**Figure 6**). In this case, the tumor sample was also positive for endothelial markers such as smooth muscle alpha-actin. Moreover, cytokeratin (AE1/AE3), indicating epithelial origin, was positive, with 8% of the cells being Ki-67-positive.

**Figure 7:**
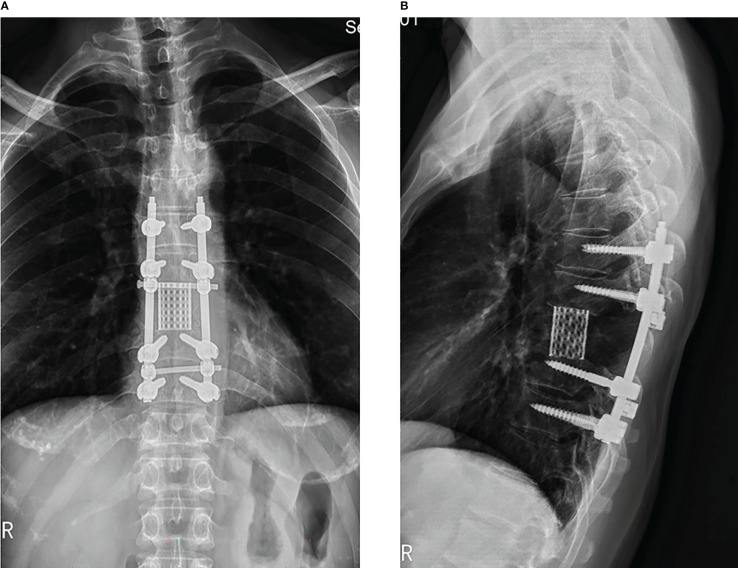
3 months follow-up after the surgery, x-ray display of thoracic spine showing transpedicular screw and a titanium mesh were used in the reconstruction of the stability of the spine in patient. (**A**: orthotopic position, **B**: side position).

During a recent postoperative follow-up after the surgery, there was no indication of any tumor progression or the emergence of any new symptoms (**Figure 7**). However, the patient and their family have politely declined radiation treatment. Currently, the patient’s back pain has been relieved, and the sensory function, including fine-touch and pinprick sensation, of the left costal arch has returned to normal.

## Discussion

Primary spinal EHE is a rare type of malignancy, accounting for only 1% of all malignant bone tumors (6). EHE is characterized by polygonal or round epithelioid endothelial cells with abundant eosinophilic hyaline cytoplasm and vesicular nuclei. Due to its local aggressiveness and metastatic potential, EHE is considered to have histological features that fall between those of hemangioma and high-grade angiosarcoma. Typically, most patients in the early stages of EHE development are asymptomatic, and the accidental discovery of the tumor during routine physical examinations is a common way to achieve early diagnosis. Although patients may not place importance on the most common symptom of atypical local pain, EHE can still lead to complications and mortality (**Table 1**). However, our study demonstrated significant therapeutic effects during the follow-up period.

**Table 1:** Complications and mortality of EHE in the references of other authors.

Author	Case number	Patient age ^&^	Segment	Therapeutic regimen	Follow-up time ^#^	Complication	Mortality	
Kitaichi, Nagai (7)	Male [8]	14–69	Lung	Surgical resection;Chemotherapy.	320	Pleural effusion; Ascites;	23.8%	
	Female [13]	15–62						
Aflatoon, Staals (8)	Male [3]	21–31	Spine	Surgical resection;Radiation therapy.	120	Postirradiation sarcoma; Back pain; Lung Metastatic disease.	12.5%	
	Female [5]	25–74						
Rosenbaum (9)	Male [4]	28–62	Lung; Liver; Bone; Soft tissue.	Surgical resection; Systemic treatment.	300	Acute myeloid leukemia; Disease.	25%	
	Female [6]							
Shiba, Imaoka (10)	Male [22]	18–78	Lung; Liver; Bone;	Surgical resection;Chemotherapy	120	Metastatic disease.	28%	
	Female [20]							
Deyrup, Tighiouart (4)	Male [28]	9–93	Head and neck; Extremities; Mediastinum; Trunk; Genitals and retroperitoneum.	Surgical resection;Chemotherapy; Radiation therapy.	60	Metastatic disease.	19%	
	Female [21]							

^&^ represents years; ^#^ indicates months.

Pathological examination is considered the gold standard for EHE diagnosis. In this case, we performed immunohistochemistry, and the examination results were consistent with diagnosis of EHE. However, with advancements in medical technology, new approaches such as molecular genetic testing can be applied to further clarify the diagnosis of EHE. A specific recurrent WWTR1-CAMTA1 gene fusion has been detected in the majority of EHE patients, and this genetic hallmark can be used as a new diagnostic indicator of EHE (11). Additionally, the detection of the WWTR1-CAMTA1 gene fusion can distinguish EHE from other benign or malignant vascular tumors (11, 12). Some recent studies have suggested that an alternate YAP1-TFE3 fusion, found in a subset of cases, may also be meaningful in EHE diagnosis. However, it remains unclear whether the YAP1-TFE3 gene fusion should be used to identify EHE (12). In the present case, preoperative puncture biopsy, clinical symptoms, and imaging features suggested that the patient required surgical intervention. Therefore, we performed TES, which resulted in satisfactory clinical results. We also recommended that the patient and his family undergo genetic testing. However, although we suggested genetic testing, the family members refused, and we believe that this did not affect the patient’s treatment and prognosis.

There are no standard treatment courses available for the management of spinal EHE. Preoperative embolization and surgical resection, followed by postoperative radiotherapy and/or chemotherapy, are the alternative options. Surgical resection is generally considered the optimal treatment approach (13). However, en-bloc resection of spinal EHE can be challenging due to the highly vascular nature of the tumor and the risk of intraoperative bleeding. The extent of surgical can range from wide margin to marginal or intralesional resection. Several clinical studies suggest that wide resection (i.e., TES), should be performed to achieve favorable outcomes (14). Luzzati et al. reported the largest case series of ten spinal EHE patients and found that patients who received relatively broad or marginal resection had better prognoses (15). While TES is currently considered the preferred choice to control spinal EHE progression, further analysis of large volumes of clinical outcomes and substantial experimental data is needed to establish the ultimate conclusion. In our case, we successfully performed a wide resection and achieved a clear resection margin.

In addition to surgery, systemic therapy, including chemotherapy and radiotherapy, may also benefit patients with spinal epithelioid hemangioendothelioma (EHE) According to a few reports, radiotherapy for partially excised spinal EHE lesions can be effective (8). Moreover, some clinical centers use radiation (45–50 Gy) as a postoperative systemic treatment to achieve long-term local control and pain relief (16). While systemic therapy can provide physiological support for patients who refuse other treatments (17, 18), surgery remains the optimal treatment choice. However, there is currently no evidence to support a strong correlation between chemotherapy or radiotherapy and good prognosis in patients with spinal EHE. In our case, the patient declined radiation or chemotherapy treatment, and we followed the patient for 3 years without observing tumor recurrence. We plan to extend the follow-up period if necessary. Our patient, who received only extensive resection surgery with favorable outcomes, may provide insight for developing a standardized treatment plan for EHE in the future. In contrast, expectant observation may be an alternative approach for unresectable tumors or those that may lead to severe complications if resected.

Regarding prognosis, patients with multifocality, pleural involvement, lymph node invasion, or remote metastases generally have considerably poor outcomes. Conversely, patients without these adverse factors have an average 5-year survival rate of >70% (9). In this case, only one lesion was presented, and there was no evidence of multifocality, pleural involvement, lymph node involvement, or metastasis. According to Rosenbaum et al. (9), satisfactory clinical outcomes can be achieved after standard surgical treatment and 3 years of tumor-free survival. However, according to recent research, the WWTR1-CAMTA1 gene fusion may indicate that other genetic anomalies can lead to more aggressive biological behavior (9). Simultaneously, the 5-year survival for WWTR1-CAMTA1 fusion is 59%, compared to 86% for YAP1-TFE3 fusion (9). Some asymptomatic patients may also have a good prognosis through careful monitoring, as spontaneous regressions have been reported (7). Unfortunately, our patient declined the proposal for genetic testing. We believe that genetic detection is significant for the prognosis of EHE patients and for EHE prevention in their families. Screening for the gene mutation causing EHE in other family members can enable early diagnosis and prompt treatment, particularly for asymptomatic family members, to prevent distant metastases and poor prognosis. The prognosis of EHE varies depending on anatomical site. EHE of soft tissues can be indolent. However, EHE arising in the lung or bone have a worse prognosis than soft tissue tumors, and patients often present with metastatic disease. Additionally, in the recently identified YAP1-TFE3 subset of cases, the metastatic rate may be higher. A traditional risk stratification is based on mitotic activity and tumor size. During the one-year follow-up, the patient reported remission of back pain and no recurrence of pain.

This case report has several limitations that should be acknowledged. The follow-up period is less than 5 years, and the study involves only one patient. Therefore, to accurately evaluate the therapeutic effect of TES, multicenter, large-sample, long-follow-up randomized controlled trials are necessary. Additionally, the patient did not undergo genetic testing, which limits the ability to make definitive conclusions regarding the impact of genetic anomalies on the clinical outcomes of EHE. As such, genetic counseling and education should be offered to patients and their family members for prevention, early diagnosis, and treatment.

## Conclusion

TES has been demonstrated to be safe, effective, and reliable for the treatment of EHE, and in this case, we were able to successfully diagnose and treat the patient. Nonetheless, the patient requires long-term follow-up to assess the clinical outcome and to determine if any recurrence or metastasis occurs. This is especially important given that EHE is known to have a variable clinical course and prognosis, which can be influenced by a range of factors such as genetic anomalies, tumor location, and disease stage. Long-term follow-up will also provide additional insight into the efficacy and safety of TES as a treatment modality for EHE. This study was reported in agreement with principles of the CARE guidelines (19).

## Data availability statement

The datasets used and/or analyzed during the current study are available from the corresponding author on reasonable request.

## Ethics statement

Ethical review and approval was not required for the study on human participants in accordance with the local legislation and institutional requirements. The patients/participants provided their written informed consent to participate in this study. Written informed consent was obtained from the participant/patient(s) for the publication of this case report.

## Author contributions

Data Curation: Writing - Original Draft: WG, YQ. Writing - Review and Editing: SZ. Visualization: TH, JZ, LY. Supervision: LC. Project administration: QL, JL. All authors contributed to the article and approved the submitted version.
