# Corrigendum: Case Report: Surgical treatment of a primary giant epithelioid hemangioendothelioma of the spine with total en-bloc spondylectomy

**DOI:** 10.3389/fonc.2023.1308503

**Published:** 2023-11-09

**Authors:** Wanbao Ge, Yuan Qu, Tingting Hou, Jiayin Zhang, Qiuju Li, Lili Yang, Lanqing Cao, Jindong Li, Shanyong Zhang

**Affiliations:** ^1^ Department of Spine Surgery, Second Affiliated Hospital of Jilin University, Changchun, Jilin, China; ^2^ Department of Pathology, Second Affiliated Hospital of Jilin University, Changchun, Jilin, China; ^3^ Department of Thoracic Surgery, Second Affiliated Hospital of Jilin University, Changchun, Jilin, China

**Keywords:** epithelioid hemangioendothelioma, total en-bloc spondylectomy, vertebral lesion, vascular neoplasm, giant

**Error in Author List**

In the published article, there was an error in the author list, Wanbao Ge and Yuan Qu share first authorship, and this was erroneously omitted in the published. The corrected author list appears below.

Wanbao Ge1(co-author), Yuan Qu1(co-author), Tingting Hou1, Jiayin Zhang1, Qiuju Li, Lili Yang1, Lanqing Cao2, Jindong Li3 and Shanyong Zhang1*

The authors apologize for this error and state that this does not change the scientific conclusions of the article in any way. The original article has been updated.

